# iNICU – Integrated Neonatal Care Unit: Capturing Neonatal Journey in an Intelligent Data Way

**DOI:** 10.1007/s10916-017-0774-8

**Published:** 2017-07-26

**Authors:** Harpreet Singh, Gautam Yadav, Raghuram Mallaiah, Preetha Joshi, Vinay Joshi, Ravneet Kaur, Suneyna Bansal, Samir K. Brahmachari

**Affiliations:** 1 Academy of Scientific and Innovative Research, New Delhi, India; 2 Oxyent Medical Private Limited, 801 DLF Tower B, New Delhi, 110025 India; 3 Kalawati Hospital, Shiv Chowk, Rewari, Haryana India; 4 Fortis Le Femme, Greater Kailash -2, New Delhi, India; 5 Kokilaben Dhirubhai Ambani Hospital, Mumbai, India; 6 CSIR-Institute of Genomics and Integrated Biology, Mathura Road, New Delhi, 110020 India

**Keywords:** Neonate health, Neonatology intensive care unit, PACS, EMR/his, Deep learning, IoT cloud

## Abstract

Neonatal period represents first 28 days of life, which is the most vulnerable time for a child’s survival especially for the preterm babies. High neonatal mortality is a prominent and persistent problem across the globe. Non-availability of trained staff and infrastructure are the major recognized hurdles in the quality care of these neonates. Hourly progress growth charts and reports are still maintained manually by nurses along with continuous calculation of drug dosage and nutrition as per the changing weight of the baby. iNICU (integrated Neonatology Intensive Care Unit) leverages Beaglebone and Intel Edison based IoT integration with biomedical devices in NICU i.e. monitor, ventilator and blood gas machine. iNICU is hosted on IBM Softlayer based cloud computing infrastructure and map NICU workflow in Java based responsive web application to provide translational research informatics support to the clinicians. iNICU captures real time vital parameters i.e. respiration rate, heart rate, lab data and PACS amounting for millions of data points per day per child. Stream of data is sent to Apache Kafka layer which stores the same in Apache Cassandra NoSQL. iNICU also captures clinical data like feed intake, urine output, and daily assessment of child in PostgreSQL database. It acts as first Big Data hub (of both structured and unstructured data) of neonates across India offering temporal (longitudinal) data of their stay in NICU and allow clinicians in evaluating efficacy of their interventions. iNICU leverages drools based clinical rule based engine and deep learning based big data analytical model coded in R and PMML. iNICU solution aims to improve care time, fills skill gap, enable remote monitoring of neonates in rural regions, assists in identifying the early onset of disease, and reduction in neonatal mortality.

**Electronic supplementary material:**

The online version of this article (doi:10.1007/s10916-017-0774-8) contains supplementary material, which is available to authorized users.

## Introduction

Globally 15 million babies are born preterm (before the completion of normal 37 weeks of gestation) every year i.e. more than 1 in 10 babies is born preterm [1]. In India alone, 23% (>3.5millions) of preterm births are annually reported [1]. These babies are extremely vulnerable and usually die due to several birth complications, acquired infections and damage to their brain, lungs or eyes. Preterm birth complications account for 0.748 million deaths per year in India which is 26% of the world’s neonatal deaths [2]. In India, major causes of preterm deaths are pre-maturity/ preterm (35%), neonatal infections (33%), intra-partum related complications/ birth asphyxia (20%) and congenital malformations (9%) [2]. Preterm babies who are able to survive would remain immune challenged and are highly prone to learning, hearing and visual disabilities [3]. Orders of severity and complications in preterm birth are usually inversely associated with gestational age that has 3 categories: extremely preterm (<28 weeks), very preterm (28 to <32 weeks) and moderate to late preterm (32 to <37 weeks). Neonates born in <32 weeks of gestation are grouped under “critical care group” and need to be maintained in intensive care units specialized in the care of ill or premature newborn infants, termed as NICU. Based on the criticality of the newborns, there are 3 defined levels of NICU: Level I stabilize sick newborns and low-birth weight babies, which do not require intensive care. Level II are equipped to support sick newborns other than those who need ventilator support and surgical care. The level III units are the neonatal intensive care units (NICUs) [4].

In last decade, India has seen a decline in the neonatal mortality but observed rate was found to be slower than the expected [5]. In 2011, Govt. of India has recognized the need of further expansion of neonate care centers by 30–50% [6]. Thus, Ministry of Health & Family welfares developed 448 new Sick Newborn Care Units (SNCU) with level 2 facility containing a total of 6408 beds which has supported >6,00,000 babies by 2013 [7]. The number of trained manpower with proper infrastructure is still daunting need in NICU settings [8]. NICU’s still needs more number of skilled clinical labors in order to uphold the current load of preterm births. Moreover, NICU’s requires skilled nurses in the ratio of 1:1–3 (Nurses/Patients) based on the severity of newborn’s health admitted to the NICU [9].

NICU employs several medical devices to monitor & maintain the physiological parameters of the newborn such as; ventilator, incubator, ECG monitor, blood pressure monitor, pulse oximeter, transcutaneous oxygen/carbon dioxide monitor etc. along with the infusion pump to deliver drugs and fluids. These devices produce humongous amount of proprietary data each second and store the information maximum for 72 h. For example, single ECG produces 86.4 million readings, impedance measurement by ECG leads result in 5.4 million data points, blood oxygen saturation reading gives 0.08 million data points per day for a single patient [10]. Till now lot of this crucial physiological data from these devices remain unexplored. Doctors usually consider only the maximum/average values of these physiological parameters such as, heart rate, respiratory rate etc., on hourly basis, noted manually by nurses on nursing charts/notes which also inculcates high error rate. Processing of this high frequency voluminous physiological data streams is still a big challenge but could yield significant insights to provide the quality care of neonates. Recent literature showed that physiological markers can provide early insights before the clinical signs become apparent. For example, HeRo score based on the heart rate variability with decelerations can be used as an indicator of early onset of sepsis [11]. It has also been seen that the clinical signs alone are sometimes subtle for example, apnea and feeding intolerance alone cannot be considered as the clear indicators of sepsis [12]. Thus, inclusion of physiological markers along with neonatal score and clinical signs could help in identifying the actual prognostic and/or diagnostic markers for various neonatal indications. Introduction of longitudinal translational informatics with careful workflow design incumbent with predictive algorithms could integrate manifolds of data from biomedical devices (physiological parameters), clinical documents, laboratory reports, pharmacy reports and diagnosis codes, to help in early prognosis, prevention, and diagnosis of preterm babies. However, collating diverse types of data from multiple sources itself poses few issues such as; cost, processing, storage, network bandwidth, confidentiality which could be readily addressed in current ICT setup. Thus, automation of NICU workflow by a robust big data infrastructure could facilitate monitoring and storage of the physiological and clinical phenotype parameters at various timeframes, and also help in clinical markers discovery process. Immediate benefits of implementing similar automated tool e.g. POE, has been reported to reduce transcription errors, turn-around times and timely support of results in USA [13]. This data collection can help in training semi-skilled trained manpower and improving clinical protocols in providing care to neonates in emerging countries like India.

Specialized training of clinical care people is required in order to support several challenging tasks especially associated to the care of neonates at NICU. One challenge is the rapid weight changes of the neonate’s body, which affects their nutrition demand and pharmacokinetic/ pharmacodynamics properties of drugs administered to child [14]. Thus, continuous calculation is needed and is performed manually by neonatologists to determine the appropriate nutrition and drug dosage. Moreover, the entire workflow of NICU involves the engagement of various medical experts such as, neonatologists (consultant/resident doctor/junior doctor), nutritionists, respiratory therapists, pharmacists, and specially trained nurses in corporation with ophthalmologists, microbiologists, cardiologists and surgeons. Complex concurrent care pathway flows among the interdisciplinary team in NICU are mediated by the trained clinical staff. The nurses not only follow the specialist instructions but also prepare the manual charts and reports for the hourly progress of the baby. Thus, these manual reports are highly prone to human error.

**Fig. 1:**
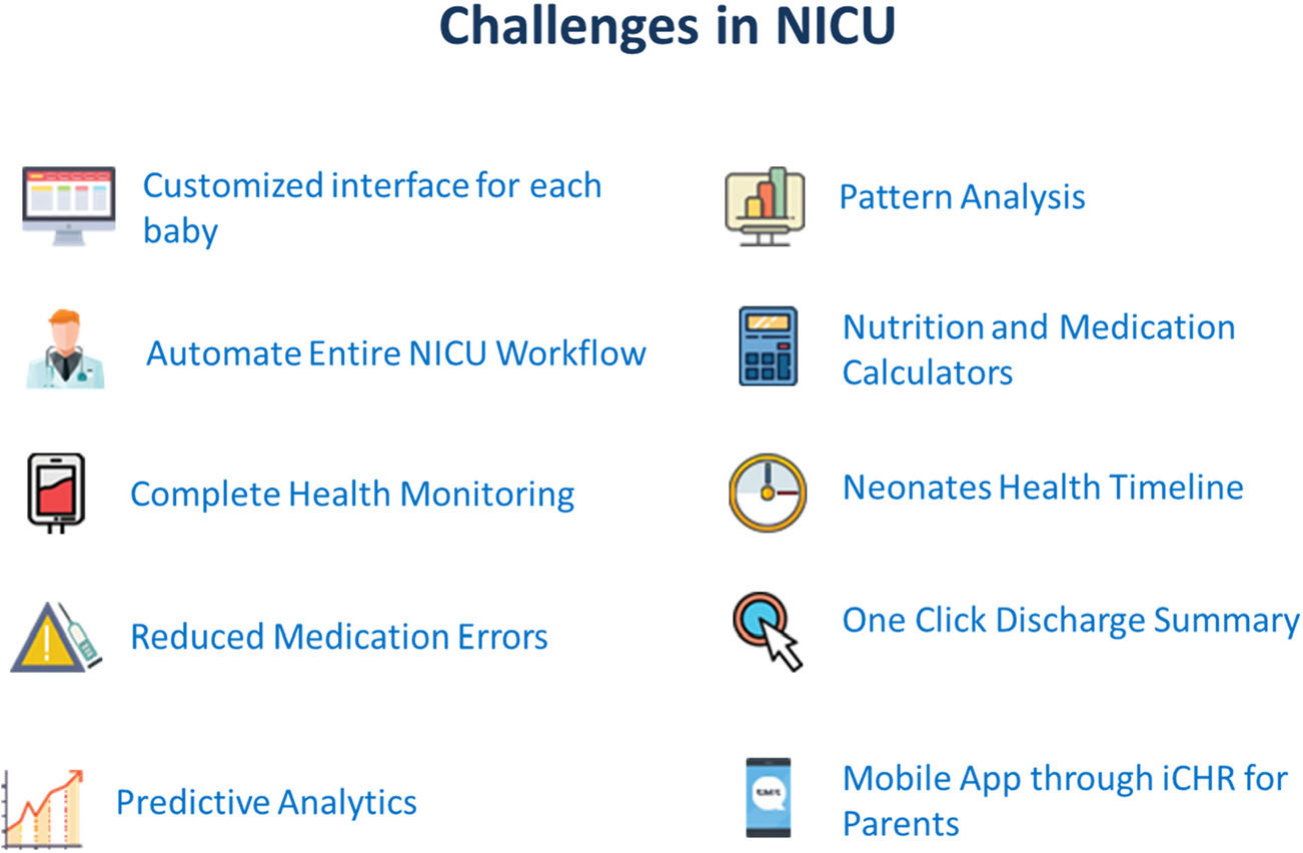
Benefits of iNICU

For achieving these goals, we are presenting in this paper, Cloud, IoT and Data Analytics based software solution, iNICU (integrated Neonatal Intensive Care Unit). iNICU is a comprehensive integrated platform especially designed to address all the current issues of NICU such as tedious workflow, integration of the data generated by multiple devices at one place, automatic drug/nutrition calculator, auto-discharge summary, complete assessment sheets for all critical biological systems of newborns, digitalized prescription, laboratory reports, nursing notes, prenatal data, notifications/alerts to the doctor, parent engagement, predictive analytics and NICU management (Fig. 1). Our vision is to provide complete automation with analytics to benchmark for the quality care of the newborns. iNICU allows concurrent real time access of multiple infants to clinical experts and thus, improves the care time. Key long term benefits of our solution are care time improvement, filling skill gap, remote monitoring of rural regions by experts, early identification of disease, and reduction in neonatal mortality. However, the scope of this article is to demonstrate the concept, development and workflow of iNICU. Field-testing of the system is ongoing in five pilot NICU sites in India. The major aim of field testing is to improve usability and standardization of assessment of various diseases in NICU environment.

## Architecture

**Fig. 2:**
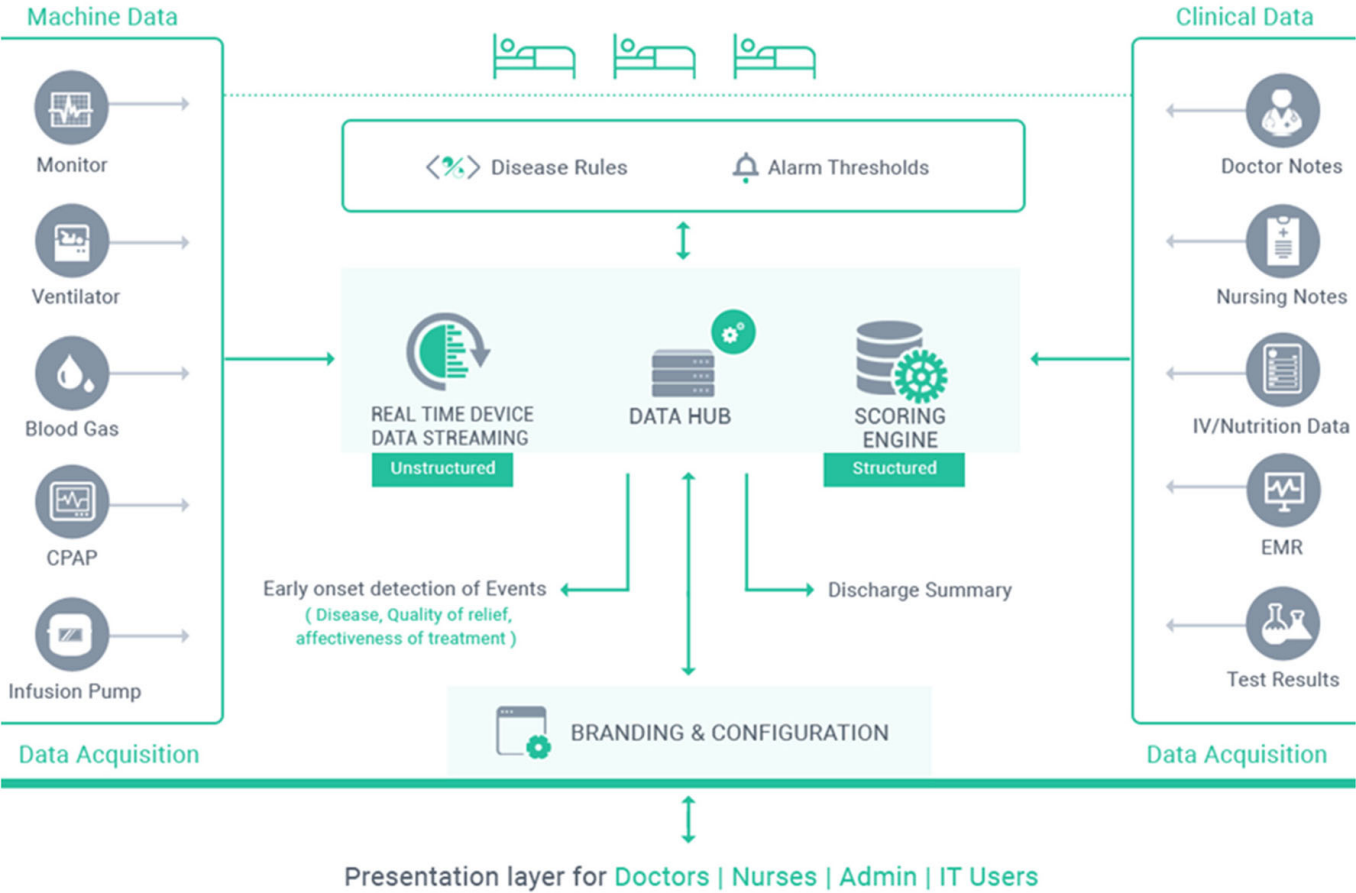
iNICU Architecture

The architecture of the iNICU system illustrated in Fig. 2, which mainly consist of three parts i.e. the machine data integration, clinical interface for iNICU and data analytics engine (consisting of data, associated clinical rules and notifications).

### Machine data integration

The machine data integration (MDI) layer is divided into two blocks a) MDI client b) MDI server. MDI client provides wrapper on HL7 and RS 232 allowing interaction of iNICU system with various devices. These devices provide data over WAN, Network or Serial cable.

Device vendors like GE, Philips, Nihon Kohden support HL7 (Health Level Protocol) protocol for data retrieval from their devices. iNICU uses open source HAPI (HL7 API) and supports HL7 2.X version to fetch data from various devices in NICU setting. Separate thread is initiated for each supporting device to acquire concurrent live data feeds. Various device specific HL7 adaptations (such as Medibus for Dragger, Intelivue for Philips and Carescape for GE) are used to establish connection with each of the medical device.

For RS 232 interface, iNICU uses RXTX Java based communication API. This is hosted on Raspberry, Arduino and Intel Edison board and placed on the acquisition device. The MDI client layer fetches data over serial port and pass acquired data to MDI server layer. Data aggregation from these devices is carried out using IoT gateway (i.e. Cybertan and Intel IoT gateway).

MDI Server layer is implemented using open source Apache Kafka. MDI Server subscribes to real time streams of data coming from various MDI client implementations. It uses Apache Cassandra to store unstructured data. MDI Server piece also integrated with Lab Information Management System via ASTM protocol (JAVA ASTM API).

### Clinical interface

The doctor/nurse interface is built using service oriented architecture. The server part is implemented using Java 1.8 language leveraging Spring Boot framework. User Interface layer is built using responsive AngularJS (JavaScript Framework) and HTML5. This allows User Interface layer to be responsive and it can run seamlessly on Tablet, Laptop and Mobile devices. JSON based REST API integration connects AngularJS and Spring layers. Patient data is accessed either from Hospital Information System as ADT (Admission Discharge Transfer) events through HL7 Integration or manually entered by the Hospital administration. Clinical data stored in PostgreSQL and Hibernate allows access of database from Java business layer. Various Neonatal calculators are coded using Drools rule engine and stored as metadata. Solution is hosted on IBM Softlayer based cloud infrastructure. Growth charts are implemented using high charts and JavaScript. Cloud component allows only HTTPS based communication protected by 256-bit encryption with web interface.

### Data analytics engine

Clinical data is stored in PostgreSQL and waveform/machine data is stored in Apache Cassandra. Normalized data was fetched from both unstructured and structured data stores. Disease based neonatal score help to categorize infant into different diseases. Incoming facts (urine output, Respiratory Rate, Heart Rate, SpO2 etc.) of child act as input to Clinical Rules and matching rules inferences are executed. These inferences generate alarm and notification which are send via SMS/Google Cloud Messaging and Apple Push Notification Service to doctor, nurses and patients (specific one).

## Highlights of iNICU system

iNICU automates the entire workflow of NICU from the admission of newborn till its discharge. It caters the entire responsibility of doctors, nurses and paramedical staff and also provides analytics for the child healthcare and hospital management system. iNICU system can be broadly classified into four important sections: 1) Digitization of clinical investigation, 2) Clinical care time improvement by auto-calculators, 3) Neonatal scores based real-time alarms, and 4) Deep learning based analytical model for early prediction of disease onset.

### Digitization of NICU workflow

#### Admission to dashboard

**Fig. 3:**
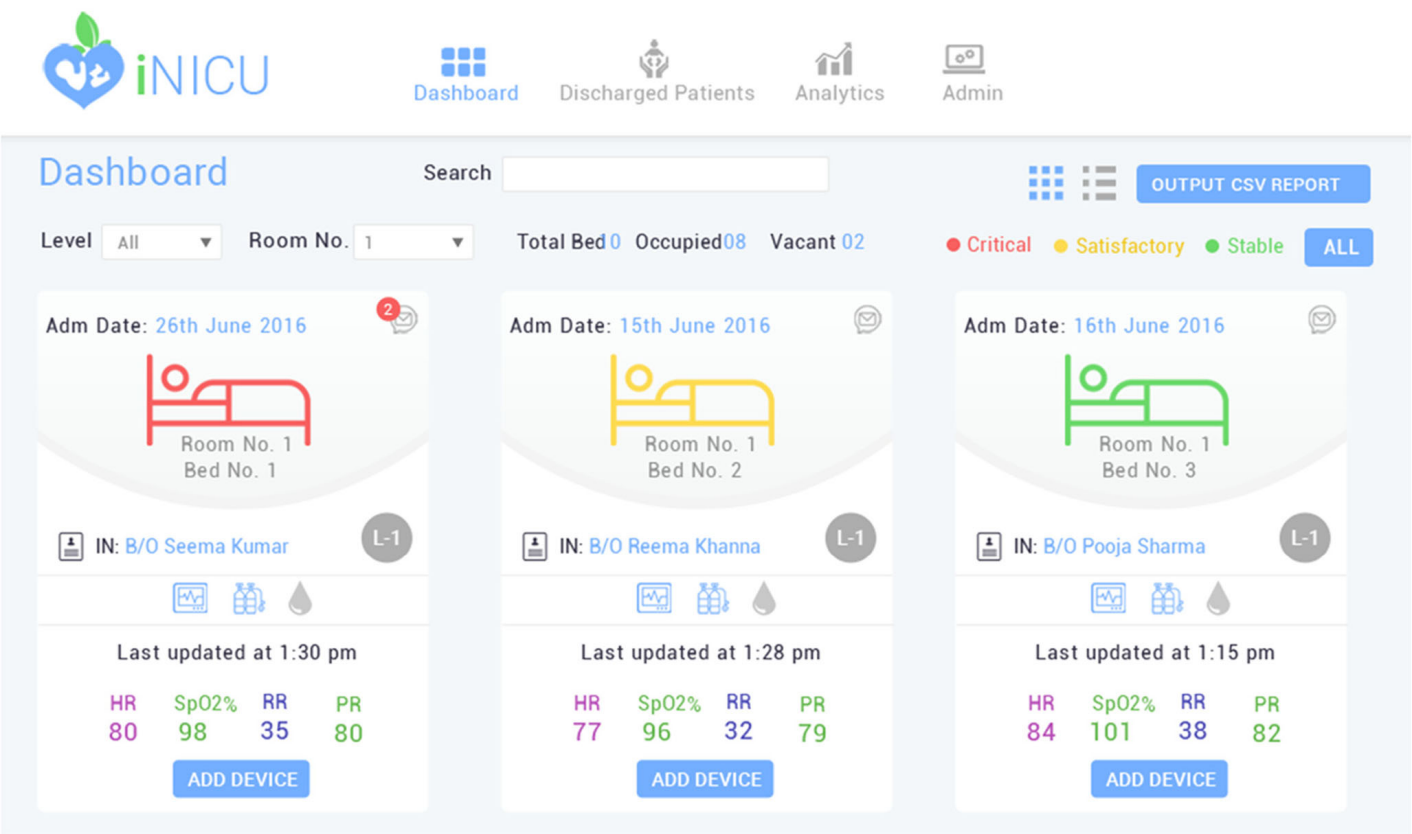
Dashboard View of iNICU

Dashboard is a collated view of NICU facility of the hospital along with the necessary personal and clinical details of the admitted newborns (see Fig. 3). Once the personal data, maternal history, birth related child data and initial assessment of child’s health have been updated in the digitized admission form, baby is admitted to the NICU/iNICU. Every admitted neonate is color-coded based on health condition i.e. critical, satisfactory and stable, so that attention could be prioritized from the first view itself. It also displays the NICU level of newborn along with the critical life supporting device parameters such as heart rate, peripheral oxygen saturation, respiratory rate and pulse rate. The dashboard gives a quick, consolidated and informative view of all neonates along with their necessary monitoring parameters and notifications/ alerts. Tabulated view in dashboard will also give a printable comprehensive table depicted weight change, feeding pattern, ventilator usage, major diagnosis (in order) for all admitted neonates.

#### Data integration- device and laboratory reports

**Fig. 4:**
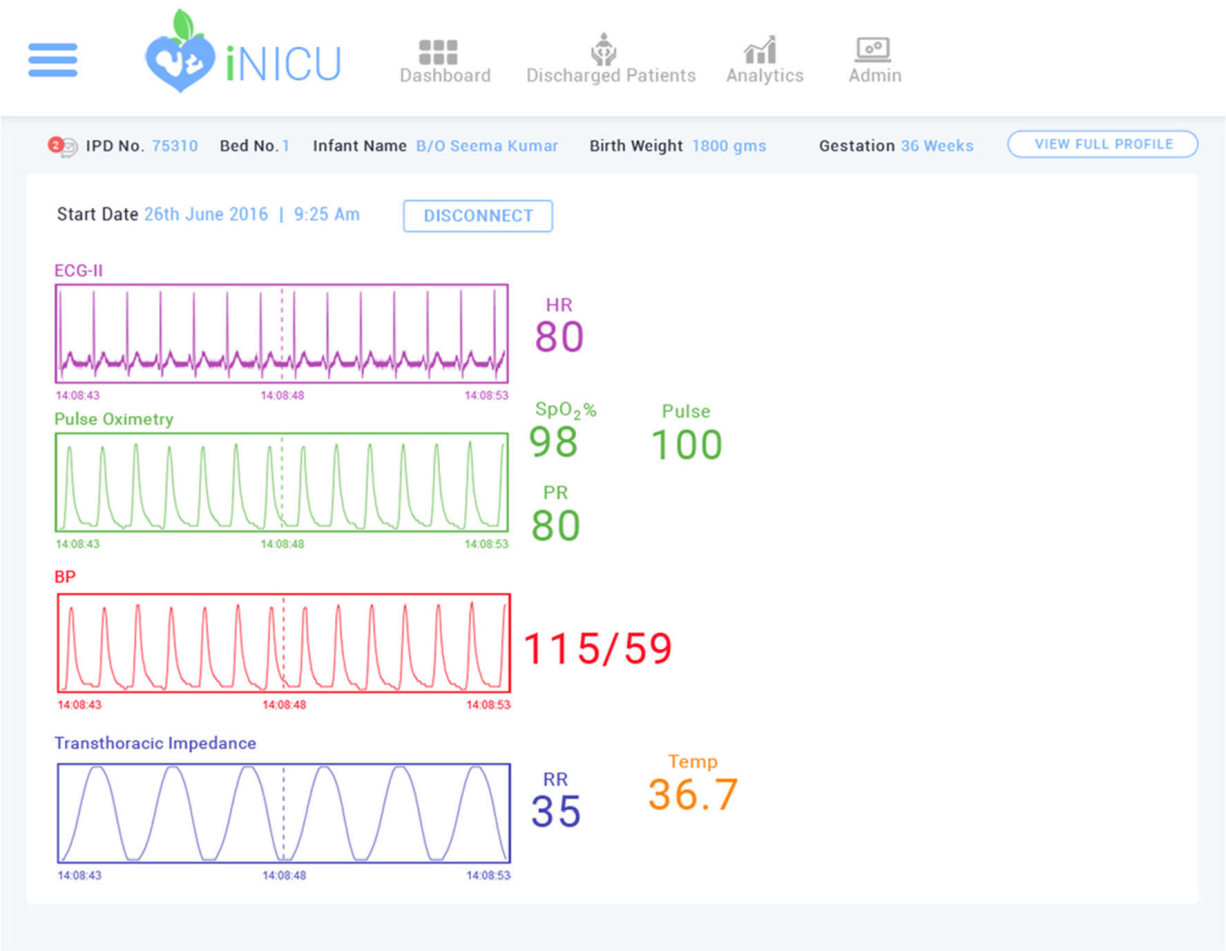
Real Time Device Data of iNICU

**Fig. 5:**
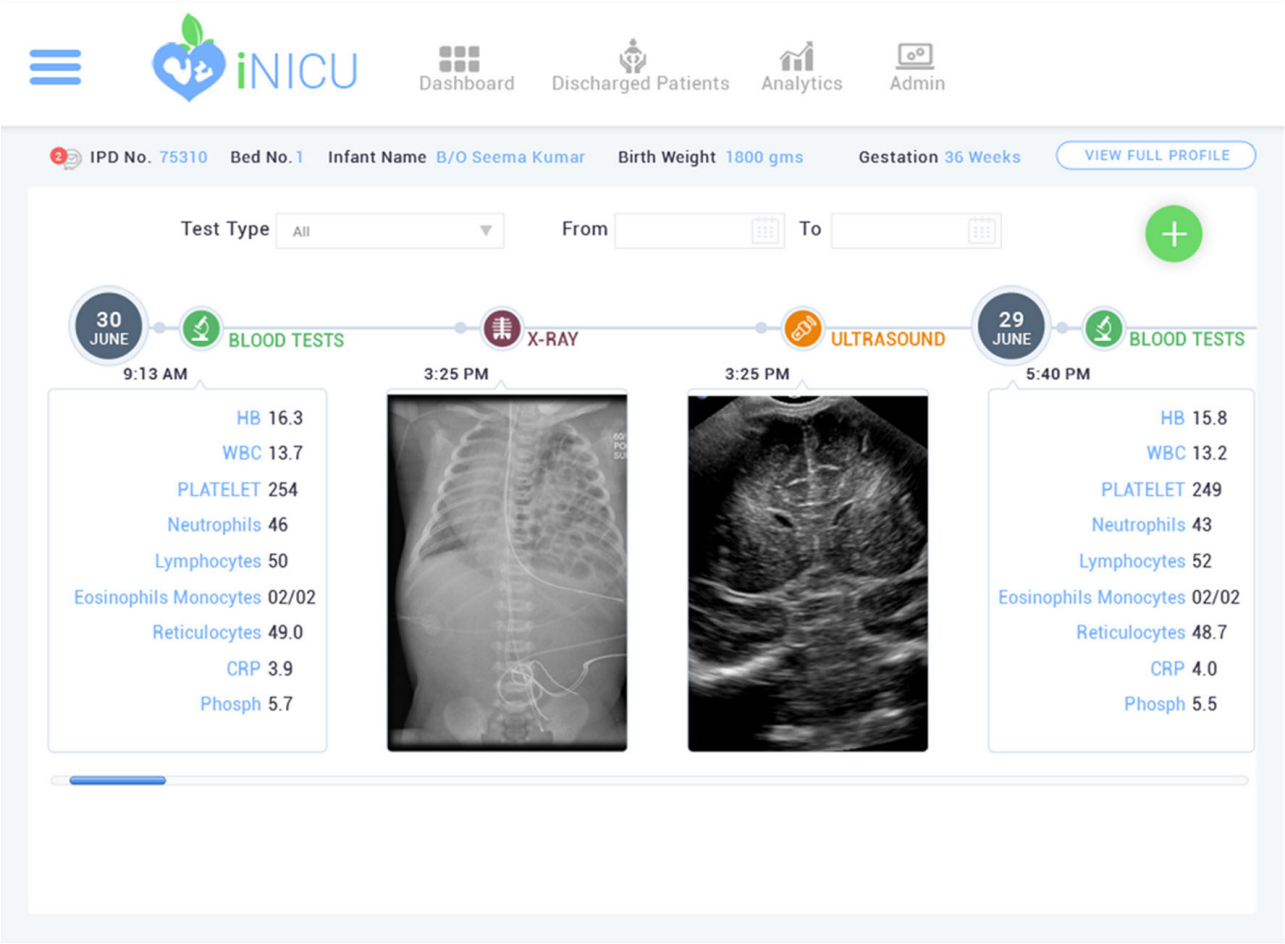
Laboratory Test Records in iNICU

Physiological streams from monitors, ventilators and blood gas machine can be viewed for every child (Fig. 4). New device can be added to iNICU using add-device module. iNICU seamlessly links with Laboratory Information Management System (LIMS) and PACS. Thus, raw data from laboratory reports as well as Image data can be viewed through iNICU (Fig. 5). Control charts visualization was used to see the trend of vital parameters such as bilirubin in neonatal jaundice.

#### Neonatologist’s assessments & nursing care

**Fig. 6:**
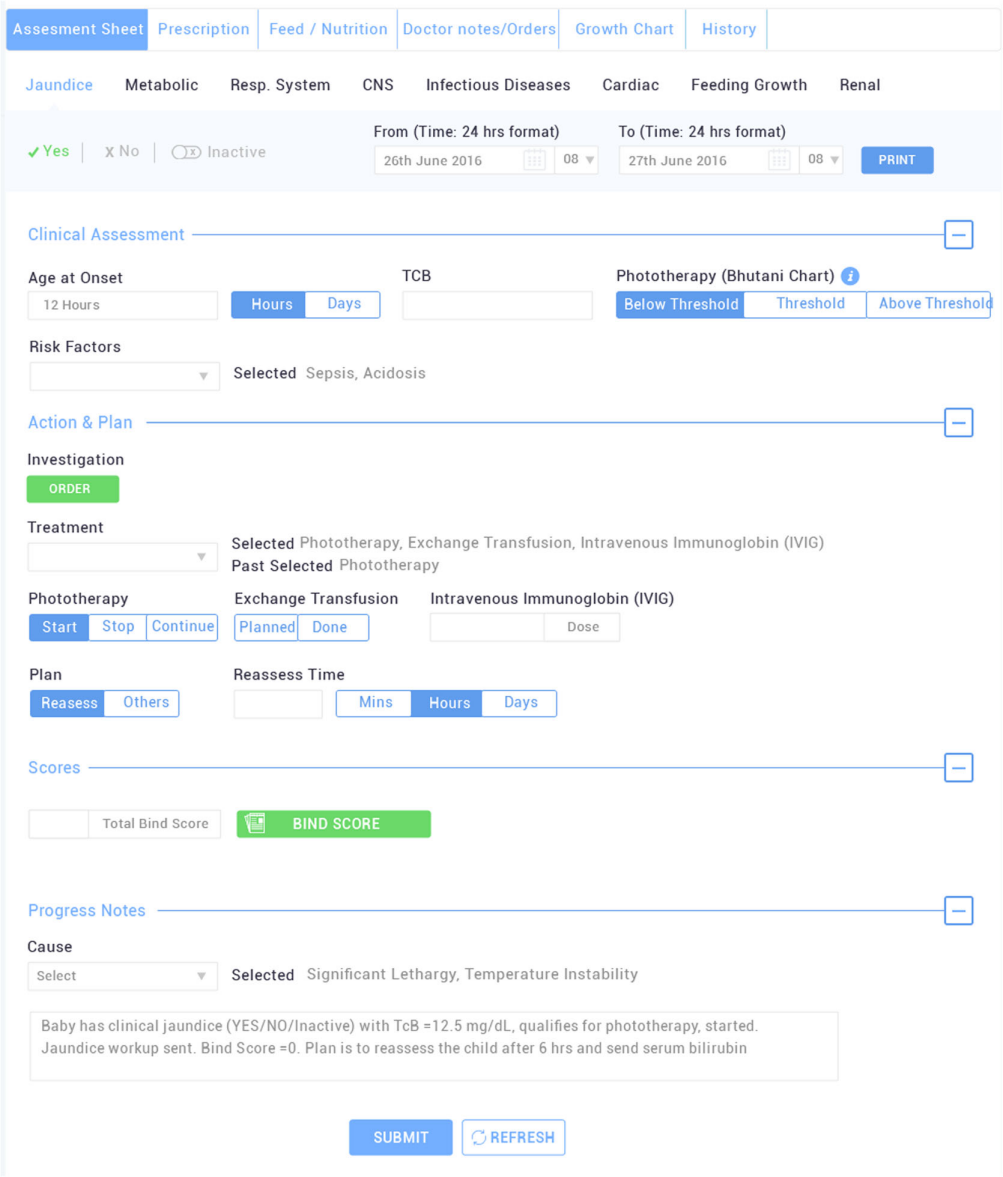
Doctor’s Panel with expanded Jaundice Assessment Screen at iNICU

Neonatologist’s panel has been split into 5 major sections: assessment sheet, prescription, feed/nutrition, notes and growth Charts (Fig. 6). Assessment sheet designed to monitor the progression and/or development of any of the known neonatal clinical events in all major biological system such as; metabolic, respiratory, infections, renal, CNS, cardiac, jaundice, malformations. Each assessment sheet was carefully design to capture the workflow of neonatologist. Each sheet has 3 panels of Clinical assessment, Action (Investigation, Medications) and Plan along with causes and associated events. Based on clinician assessment (i.e. jaundice in Fig. 6) an auto generated progress notes for each assessment entry is generated. Moreover, assessment sheet of events such as Jaundice has clinical decision recommendations like NICE (< 35 weeks of gestation) or Bhutani charts (≥ 35 weeks of gestation) for starting phototherapy or exchange transfusion. Similarly in case of hypernatremia assessment sheet, deficit calculator allows consideration of lean weight loss and insensible losses in management of sodium level. The prescription section contains the information related to medication needs of the baby such as drug name, route of intake, dose, frequency, start date, time, end date, any special comments, active and past medication record. The baby feed can either be given as expressed breast milk (EBM), formula milk, EBM supplemented with human milk fortifier lactodex (HMF) or intravenous fluids (IVFs) as decided by the doctor. Neonatologist defines the mode, time interval and volume of the feed to be given to the baby in the Feed/Nutrition section. Doctor notes are designed for doctors to incorporate diagnosis, issues, plans and special notes as free text. Growth curves are the dynamic curves used to give the instant view on the advancement of growth parameters of the infant as per the Fenton’s standard (weekly basis). Thus, sudden drop or rise in the height, weight and head circumference of the newborn can be noted and immediate attention could be provided. These instructions are linked to nursing notes which gets auto populated, thus enforcing the nurses to follow the doctor’s advice without any error.

Nursing notes are observatory notes to capture hourly and/or daily clinical observations of neonates by the nurses. Measurement of height, length and head circumference of the baby is captured daily and any weight gain/loss would be auto populated and notified. Nursing notes also capture derived value of vital physiological parameters from the medical devices. Fluid intake and medication details with any added instructions are auto-populated from the doctor’s section and generate periodic reminders for the prescribed medication. Head-to-Toe assessment, physiotherapy, phototherapy if recommended can be monitored and recorded by the nurse in their respective sections. These simple separate observatory sheets for various different needs and auto-filled instructions/reminders provide a holistic and hassle free interface to the nursing staff thereby minimizing the possibility of any error.

#### Discharge summary & hospital analytics/management

All personal information, maternal history, child details at birth, doctor’s notes with issues, diagnosis and medication details get auto-populated to create a complete discharge summary of a neonate that can be downloaded in just one click. List of all the discharged patients can be accessed through the discharged patients tab along with his/her discharge summary. Tracing the entire medical history of any admitted newborn become much easier and safely maintained in the software.

Hospital analytics allows administrator to see division of patients across different levels of NICU, infection rate present in NICU (compare to standard), mortality score, human breast milk consumption score and death counts due to major NICU diseases like sepsis, NEC and asphyxia.

### Clinical care time improvement: Neonatal calculators

**Fig. 7:**
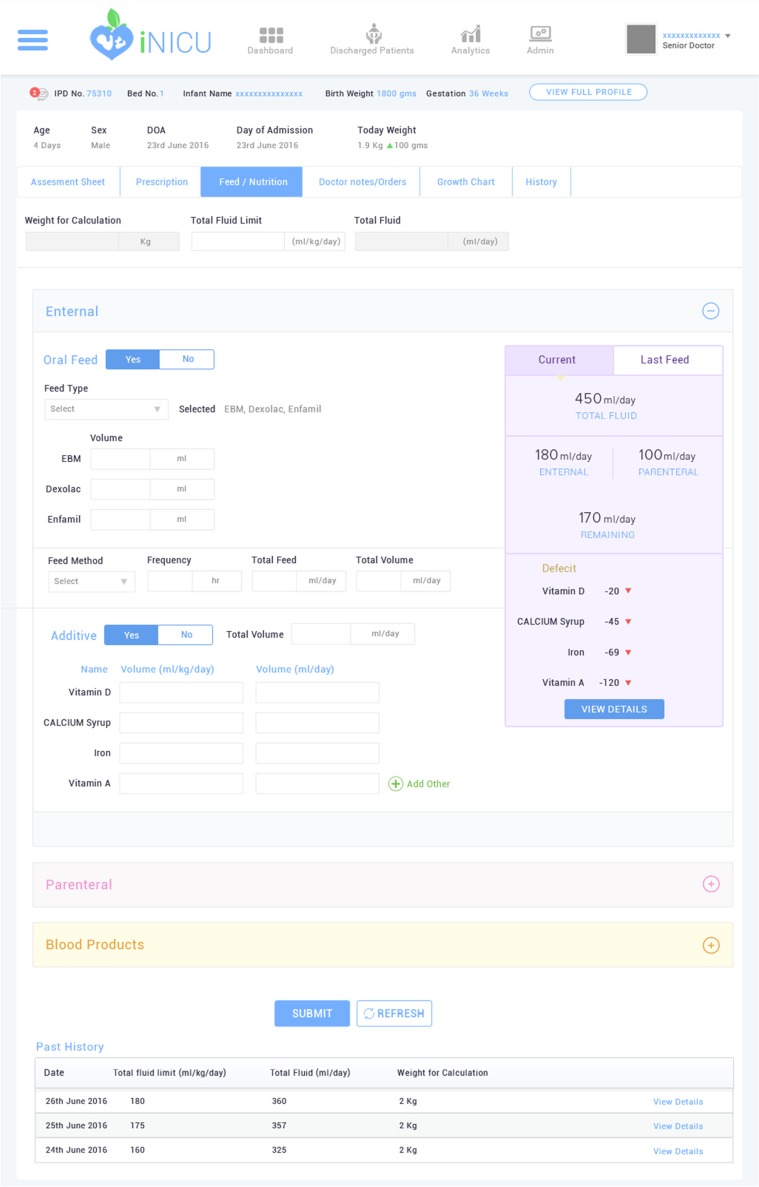
Nutrition Calculator Screen at iNICU

iNICU improves clinical care time by integrating several key calculators used in the neonatal care such as, gestation calculator, dose Calculator, nutrition calculator (EN and PN), calorie calculator, dextrose calculator. Total fluids requirements vary as per the gestational age and weight of the baby [15]. Nutrition requirement needs to be computed in a step-wise orderly manner for feed, medication, IV fluids (dextrose/salts) and blood transfusion, if any, on daily basis (Fig. 7). However, preterm and critically ill neonates (usually under 1200 g) are unable to receive normal feed by mouth. Total Parenteral Nutrition (TPN) is then used to deliver medication and all essential nutrients intravenously to the newborn [16]. TPN includes mixture of fluids, electrolytes, sugar, amino acids (proteins), vitamins, minerals, and lipids. Nutrition calculator of iNICU auto-computes daily nutritional needs of neonate. iNICU also maintains the entire record of the feeds consumed by the infants. Also, iNICU drug dose calculator auto-computes and auto populates the correct volume/ amount of drug as per the current weight of baby (Supplementary Fig. 1).

### Neonatal scores

Neonatal Scores are statistical indexes, which can reproducibly predict mortality and specific morbidities in the neonates. These scores are well accepted among major clinical communities for the risk assessment of newborn at NICU [17]. iNICU has integrated nine neonatal scores to support the clinicians in physiological system assessment of each of the newborn. For example; APGAR [18] and BALLARD [19] are used to predict the overall status of the physical and neuro muscular activities of the infant, respectively. Others scores implemented in iNICU are; Downes [20] and Silverman Anderson for respiratory distress syndrome [21], Bell’s staging for metabolic systems [22] and Bind Score for Jaundice [23], HIE scores by Sarnat & Sarnat for CNS [24], Rodwell for sepsis [25] and Volpes for heart’s condition [26].

### Analytics for disease onset prediction (Deep learning neural network)

**Fig. 8:**
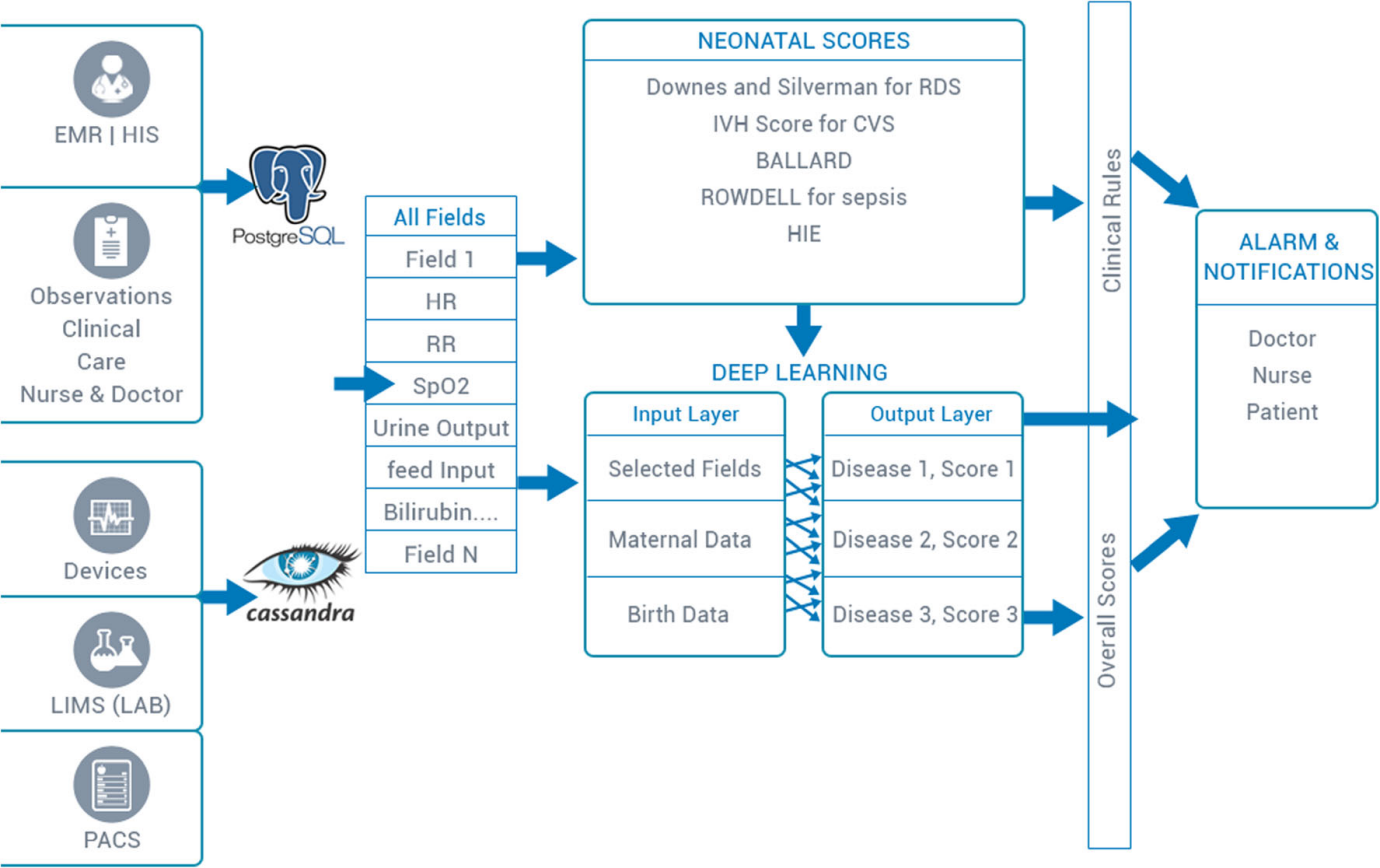
Overview of iNICU Analytical Framework

Although above statistical scores help the clinicians in predicting the disease state of neonates, there is a need of more robust and improved analytical model that could utilize the multiple data fields with high accuracy [27, 28]. Multiple medical fields have been clustered into two buckets of structured (EMR/HIS and observatory fields from Doctor’s & Nursing notes) and unstructured data (medical devices integration, LIMS and PACS) (Fig. 8). These two buckets completely capture the vital signs, clinical observations, physiological derived data/waveforms, imaging data, microbiology reports that represents the overall health status of the neonate. From the data hub, all these fields are captured to compute existing consensus based neonatal scores as discussed in above section to build clinical rules engine. Predicted diseases generate alerts for the hospital staff. Until date, iNICU captures 136 fields excluding laboratory data (Supplementary Table 1). Essential features based on fields used in neonatal scores, maternal history and birth related data are fed as input layer to Neural network. The output layer is build using disease identification (from systematic assessment) and doctor notes. An aggregate health status score has also been computed using disease severity index which used to raise alerts for doctors. Using pilot data, various case-control cohorts for above neonatal diseases will validate the predictive physiological models. Currently, iNICU is the first and the only such initiative present in the Indian health industry and has already been deployed at four major hospitals having a total of >50 NICU beds for the pilot run.

Since iNICU data acquisition is currently ongoing, we have leveraged publicly available critical neonatal dataset MIMIC-III (Medical Information Mart for Intensive Care) used to build disease specific models leveraging deeplearning4j technology [29]. MIMIC-III comprises 7863 neonates with 1576-recorded vitals (Supplementary Table 2). However, iNICU currently captures 136 essential vitals (excluding lab results). MIMIC III vitals can be majorly characterized as observational, physiological and laboratory related data. Out of >1500 reported vitals, 34 seems to be crucial for newborn as it is being observed in >70% of the data. Also, top 11 fields are mandatory observatory fields that need to be recorded once for most of the patients. Few common physiological fields such as HR, RR, SpO_2_ which is captured with high frequency per patient. Standard laboratory vitals such as hematocrit, platelet, RBC etc. are captured frequently. Other infrequent vitals seem to be disease or condition specific and thus, need not be recorded for every admitted newborn.

Newborn data has total 872 icd9_codes used for the billing. Supplementary Table 3 represents these icd9_codes with their patient frequency. ICD 9 code vs patient frequency was utilized to select top 4 disease conditions which are respiratory problems, jaundice, prematurity and heart problems, for neonate population which accounts for 56% (4399) of data.

## Limitations & challenges

iNICU is designed to automate NICU workflow with user friendly design and features for doctors and nurses. In resource crunch settings in India, care of a child takes precedence over entering the longitudinal clinical care data by intensivist. iNICU system allows saving of data in retrospective manner for analytics which helps to reduce neonatal mortality and morbidity. Major architectural challenges addressed in iNICU were: 1) capturing entries with minimal chances of error and 2) capturing the multi-dimensional clinical electronic patient records from lab, devices and other third party systems. To overcome these, we have designed time series (longitudinal) database based on different neonatal episodes/events. Major neonatal events are classified as separate assessments linking the relevant laboratory tests, device parameters, and interventions to identify clinically relevant features. In addition, relationships between events and result of interventions are well captured through automated progress notes. To overcome clinician difficulty in pattern recognition from physiological data, we have pre-computed scores e.g. PhysiScore which could predict the morbidity status of the neonates based on physiological streams for first 3 h of life [30].

To enhance iNICU usability, we provide clinicians various clinical recommendations such as NICE or Bhutani charts for phototherapy/transfusion in Jaundice, daily recommended diet values from ESPGHAN etc. Auto-generated daily progress notes, inbuilt calculators and less typing will reduce the clinician’s efforts and increase their care time. Assessment scores are accompanied by pictures and guidelines (e.g Ballard) to assist the clinicians.

System generates reminders based on defined clinical guidelines i.e. BPD classification based on 28 days of ventilator usage for preterm babies at 36 weeks. However, physiological data based alarms (i.e. apnea, bradycardia and desaturation) need to be validated by nurses/doctors as a check for any device artifact. To ensure data completeness or avoid incorrect entry, we have various validations such as; pop-up reminders for mandatory fields, mostly auto-filled data from lab/device if possible, only allowable ranges in drop down. We also have user manuals and training sessions for doctors and nurses.

To handle issues related to power outage and network connectivity, iNICU system deployment in rural areas allows offline-working capability. Server residing in local network is regularly synchronize with cloud infrastructure based on availability of services.

iNICU also captures quality indicators such as VAP, CLABSI, antibiotic and ventilator usage which allows the NICU units to monitor and compare their NICU performances. Vital parameters are provided in tabular, line series and control chart visualizations. Each NICU sites has its implementation and control technique. Additionally feedback from each NICU unit to further improve the usability is being incorporated.

## Conclusions

iNICU is a cloud-based solution leveraging IoT and Big Data, connecting the generated source data from various devices, LIMS and EMR/HIS. It’s a cumulative repository to assimilate and disseminate neonate health information. This application is designed to automate the responsibilities of various roles/owners (Nurse, Resident Doctor, Senior Doctor, Pediatrician, and Administrator). It has an interacting interface that captures the details for every neonate and virtually eliminates human error. Continuous monitoring, data recording and notifications related to every neonate are easily accessible through the cloud-based application. All essential parameter of a neonate’s progress like growth charts and automatic calculators e.g. nutrition is provided to reduce manual effort to trace them on paper and thereby reduce care time. Clinical rule engine and Deep learning based analytical model would ensure early onset of disease prediction and thus, reduce infant mortality. American Academy of Pediatrics (AAP) has reported critical functional areas that need to covered for complete pediatric care such as immunization management, growth tracking, medication dosing, and patient identification [31]. Thus, iNICU data is extended into integrated Child Health Record (ICHRCloud) application, which is an engagement platform to record and monitor the immunization schedule, health, growth, and developmental profile of the child from birth to the age of 20 years. Thus, iNICU with iCHR cloud would provide complete growth surveillance of the child. iNICU/iCHR cloud has also been recognized as one of the best innovative solutions to three nationwide challenges in 2016. 1) Smartcamp challenge for Health Tech organized by IBM in association with T-Hub, 2) Innovate for Digital India Challenge (IFDI) 2.0 by Intel and Department of Science and Technology, Government of India and 3) Centre for Innovation Incubation and Entrepreneurship Healthcare Accelerator program anchored at IIM-Ahmedabad. In near future, we plan to link our iNICU solutions at various sites into NICU Analytics as a Service (NICaaS), a grid which will bridge the gap of specialized doctors for NICUs across the country by providing services in the field of Neonatology with a network of remotely connected NICUs. It will act as a grid across the country allowing neonatologists connected with the tertiary care centers. This ensures expert advice for acute diagnosis and early intervention for premature neonates in NICUs. This will enhance the reachability and save the time of neonatologists without compromising on quality care for neonates.

## Electronic supplementary material

ESM 1 (DOCX 67 kb)

ESM 2 (XLSX 58 kb)

ESM 3 (XLSX 36 kb)
