# Supplementary material for: iNICU – Integrated Neonatal Care Unit: Capturing Neonatal Journey in an Intelligent Data Way
Source: J Med Syst. 2017 Jul 26;41(8):132. doi: 10.1007/s10916-017-0774-8 (PMC5529490; doi:10.1007/s10916-017-0774-8)
Supplement: Supplementary file 1 — (DOCX 67 kb) [file 10916_2017_774_MOESM1_ESM.docx]

**iNICU – Integrated Neonatal Care Unit: capturing neonatal journey in an intelligent data way**

Authors: Harpreet Singh^1,^^2^, Gautam Yadav^3^, Raghuram Mallaiah^4^, Preetha Joshi^5^, Vinay Joshi^5^, Ravneet Kaur^2^, Suneyna Bansal^2^, Samir K. Brahmachari^1,6^

^1^*Academy of Scientific and Industrial Research, New Delhi, India*

^2^*Oxyent Medical Private Limited, New Delhi, India*

^3^*Kalawati Hospital, Shiv Chowk, Rewari, Haryana, India*

^4^*Fortis Le Femme, Greater Kailash -2, New Delhi, India*

^5^Kokilaben Dhirubhai Ambani Hospital, Mumbai, India

*^6^CSIR-Institute of Genomics and Integrated Biology, New Delhi, India*

Corresponding author: ^1,7^Samir K Brahmachari, PhD

CSIR-Institute of Genomics and Integrative Biology,

Mathura Road, New Delhi -110020, India

Email: skb@igib.res.in

Corresponding co-author: ^1,2^ Harpreet Singh

Oxyent Medical Private Limited,

801 DLF Tower B, New Delhi -110025, India

Email: harpreet_singh@oxyent.com

**Supplementary Fig. 1** Medication Calculator Screen at iNICU.

**
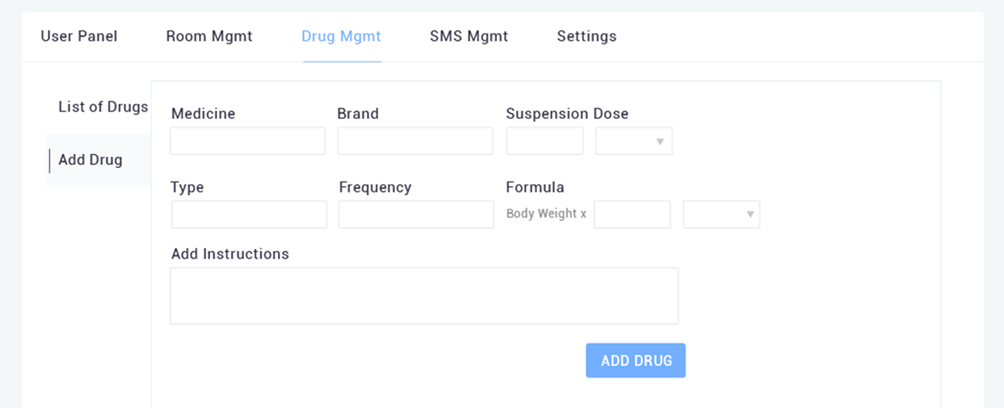
**

**Supplementary Table 1** List of Vitals captured in iNICU, excluding laboratory based parameters

| **S.No.** | **Total Vitals** |
| --- | --- |
| 1 | BIRTH WEIGHT |
| 2 | GESTATION |
| 3 | TODAY WEIGHT |
| 4 | INPUT\OUTPUT |
| 5 | FEEDS |
| 6 | AGE OF ONSET |
| 7 | DURATION |
| 8 | CAUSE |
| 9 | DCT |
| 10 | EXCHANGE TRANS |
| 11 | PHOTOTHERAPY |
| 12 | HEMOLYSIS |
| 13 | IVIG |
| 14 | HYPOGLYCEMIA |
| 15 | MINIMUM DS |
| 16 | DAY |
| 17 | MAX. GDR |
| 18 | CAUSE OF RDS |
| 19 | SPO2 |
| 20 | MAX. RR |
| 21 | APNEA |
| 22 | SURFACTANT |
| 23 | CLD |
| 24 | REASON OF MV |
| 25 | CPAP |
| 26 | AIRVO |
| 27 | NIV |
| 28 | CRIED AT BIRTH |
| 29 | POSTURE |
| 30 | CT HEAD |
| 31 | EEG |
| 32 | NNR |
| 33 | FEEDING |
| 34 | TONE |
| 35 | STAGE HIE |
| 36 | GRADE IVH |
| 37 | SEIZURES |
| 38 | MRI HEAD |
| 39 | ONSET |
| 40 | RISK FACTORS |
| 41 | PRESENTATION SYMPTOMS |
| 42 | URINE CULTURE |
| 43 | CSF CULTURE |
| 44 | ANTIBIOTICS |
| 45 | TLC |
| 46 | CRP |
| 47 | SEPSIS SCREEN |
| 48 | BLOOD CULTURE |
| 49 | ORGANISM |
| 50 | UTI |
| 51 | MENINGITIS |
| 52 | BONE INFECTION |
| 53 | PNEUMONIA |
| 54 | TYPE |
| 55 | PDA |
| 56 | ASD |
| 57 | VSD |
| 58 | SHOCK |
| 59 | INOTROPES |
| 60 | TPN |
| 61 | TROPIC FEEDS |
| 62 | FEEDING INTOLERANCE |
| 63 | CENTILE AT BIRTH |
| 64 | CENTILE AT DISCHARGE |
| 65 | RENAL FAILURE |
| 66 | MIN WEIGHT |
| 67 | ROP |
| 68 | OAE RIGHT |
| 69 | OAE LEFT |
| 70 | METABOLIC SCREEN |
| 71 | VACCINATION AT DISCHARGE |
| 72 | DAYS OF LIFE |
| 73 | LAST HEAD CURCUM |
| 74 | CURRENT HEAD CURRCUM |
| 75 | NICU DAY |
| 76 | TODAY;S WEIGHT |
| 77 | LAST WEIGHT |
| 78 | WEEKS |
| 79 | LAST LENGTH |
| 80 | CURRENT LENGTH |
| 81 | WEIGHT GAIN\LOSS |
| 82 | POSITION |
| 83 | HR |
| 84 | SKIN TEMP. |
| 85 | CORE TEMP |
| 86 | SYS BP |
| 87 | DIA. BP |
| 88 | MEAN BP |
| 89 | CVP |
| 90 | RR |
| 91 | LAX RAX |
| 92 | ETCO2 |
| 93 | SEDATION SCORE |
| 94 | GCS |
| 95 | ICP |
| 96 | CCP |
| 97 | PUPIL REACTIVITY |
| 98 | PUPIL SIZE |
| 99 | PH |
| 100 | PCO2 |
| 101 | HCO2 |
| 102 | PO2 |
| 103 | BE |
| 104 | LACTATE |
| 105 | VENT MODE |
| 106 | PIP |
| 107 | PEEP |
| 108 | PRESS SUPP |
| 109 | MAP |
| 110 | FREQ RATE |
| 111 | TIDAL VOL |
| 112 | MIN VOL |
| 113 | FIO2 |
| 114 | FLOW PER MIN |
| 115 | PPM |
| 116 | ETT (COLOR\QUANTITY) |
| 117 | FEED METHOD |
| 118 | HMF VALUE |
| 119 | FEED TYPE |
| 120 | FEED VOL |
| 121 | IV\HR |
| 122 | IV TYPE |
| 123 | AA\HR |
| 124 | LIPID\HR |
| 125 | PN RATE |
| 126 | IV TOTAL |
| 127 | BLOOD PRODUCT |
| 128 | START TIME |
| 129 | BOLUS TYPE |
| 130 | DOSE |
| 131 | NG ASPIRATE(QUANTITY\TYPE) |
| 132 | TOTAL URINE OUTPUT |
| 133 | BLOOD LETTING |
| 134 | DRAINS |
| 135 | ABD GIRTH |
| 136 | HGT |
| 137 | PHOTOTHERAPY TYPE |
